# Maternal Immune Activation and Related Factors in the Risk of Offspring Psychiatric Disorders

**DOI:** 10.3389/fpsyt.2019.00430

**Published:** 2019-06-28

**Authors:** Fiona Conway, Alan S. Brown

**Affiliations:** New York State Psychiatric Institute, Columbia University Medical Center, New York, NY, United States

**Keywords:** autism, schizophrenia, maternal immune activation, stress, microbiome

## Abstract

Maternal immune activation (MIA) at the time of gestation has been linked to increased risk of neurodevelopmental psychiatric disorders. Animal and human models have been used to evaluate the relationship between MIA and these outcomes. Given that each of these two disciplines of study have their benefits and limitations, a translational perspective is expected to illuminate more than by the use of any single approach. In this article, we discuss this translational framework and explore how it may be enhanced by the utilization of epigenetic studies and by investigating the microbiome. In this perspectives piece, we focus on the impact of epidemiologic studies, animal models, and preclinical studies in the literature on MIA as well as the potential for greater integration between fields.

## Introduction

Fetal developmental events occurring *in utero* are implicated in the postnatal health of offspring through adulthood. Environmental exposures during gestation, including maternal infection, nutritional deficiencies, toxic exposures, and other factors that cause stress during pregnancy are particularly insidious during gestation. Central nervous system (CNS) disorders, particularly schizophrenia, autism spectrum disorder (ASD), and bipolar disorder likely result from both genetic and environmental contributions. Epidemiologic studies have revealed strong connections between conditions associated with heighted maternal immune activation (MIA), resulting from infection and stress, and schizophrenia (1, 2), ASD (3, 4), and bipolar disorder (5) in offspring. At the same time, substantial advances have been made through animal models to understand the mechanisms underlying these diagnoses. More recent studies have begun to address potential mediating pathways including epigenetics, and the role of the microbiome in these disorders.

## Impact and Limitations of Epidemiologic Studies of MIA

Epidemiologic studies offer important potential inferences into etiologic processes through the direct study of human populations. Results from early ecologic studies, which focused on the comparison of groups instead of on individuals, were consistent with associations historically found between schizophrenia and prenatal exposure to influenza outbreaks (6). Later, ecologic studies, with larger cohorts and those investigating other infectious agents, showed inconsistent results and generally weak effects. However, these types of studies are imprecise in their measurement of this exposure, as individual-level data are missing. About 70% of those who were *in utero* during the influenza epidemic of 1957, but were unexposed, were incorrectly classified as exposed because ecologic studies are based on dates of birth to establish fetal exposure (7).

To address this limitation, individual-level information has been garnered through the use of birth cohort studies, by which exposures occurring during pregnancy can be documented with questionnaires, medical histories or biological samples. Individuals studied can then be longitudinally monitored for diagnoses such as ASD or schizophrenia. Birth cohort studies have shown that offspring of mothers with antibodies to certain infections, including influenza, and/or elevated C-reactive protein levels during pregnancy, were at increased risk for schizophrenia (1, 2, 8), ASD (3, 9, 10), and bipolar disorder (5, 11). This is discussed in a recent review [see Ref. (12)].

One key limitation of birth cohort studies is that they cannot be used to identify biological mechanisms underlying the pathology. The potential to draw causal inferences is further limited by potential bias and confounding, though our group and others have utilized epidemiologic and statistical approaches to reduce the impact of these factors. Since a myriad of infectious agents produce cytokines and other inflammatory markers, they can be used as a common indicator of these exposures and may operate as a shared mechanism by which neurodevelopment of offspring may be prenatally modified, thus increasing the risk for schizophrenia, bipolar disorder, and other psychiatric conditions (2, 13, 14).

## Impact and Limitations of Animal and Human Models of MIA

The abovementioned epidemiological studies have inspired research on prenatal infection and MIA using rodent and primate models. Animal models have provided unique experimental tools to overcome the limitations of epidemiological studies, such as longitudinal evaluation of neurobiological processes as well as establishing causality. They also facilitate the unraveling of cellular and molecular mechanisms, which is not possible in epidemiologic studies. Through studies of animal models, cytokines were found to act on the developing fetal brain as inflammatory signaling proteins of detrimental environmental exposures. Cytokines play critical roles in normal fetal development, including neuron proliferation, and synaptogenesis (15). However, elevated maternal proinflammatory cytokine levels cause changes in these processes and have been associated with abnormal neurodevelopment (16). The MIA models in animals allow for in-depth tracking of biologically-relevant phenotypes over time from gestation to adulthood (17). These models involve triggering the maternal immune system during fetal development using a variety of immunogens, such as lipopolysaccharide (LPS) and polyinosinic:polycytidylic acid (poly I:C), and then observing changes in the brain and behavioral development of offspring for features corresponding to human neurological disorders (18, 19). It is possible that prenatal immune challenge acts as a “disease primer” which, when combined with other environmental, genetic, and epigenetic factors, alters the trajectory of fetal neurodevelopment and may ultimately result in the emergence of a number of CNS disorders (17).

Rodents have historically been the principal species utilized in animal model studies. For example, deficits in sensorimotor gating (17, 18), depression-like behaviors (20), and high levels of repetitive behaviors (18, 21, 22) have been noted in offspring of rodent MIA models. One particular mouse study (8) reported neural and behavioral abnormalities resembling those found in schizophrenia as a result of prenatal exposure to MIA interacting synergistically with traumatizing experiences in puberty (23). Studies have also included nonhuman primates, such as rhesus monkeys (24). These models have provided greater comparability with regard to biological phenotypes and neurodevelopmental processes to humans. In one rhesus monkey model, MIA produced progeny that displayed irregular social interactions, abnormal communication, and repetitive behaviors. These results extended rodent MIA findings to behaviors that more closely mirror human behaviors, such as those in both ASD and, to some degree, in schizophrenia (25). More recently, novel evidence implicating MIA exposure with alterations of nonhuman primate dendritic morphology was found (26). This may offer insight into revealing the neuropathology of CNS disorders related to MIA and pave the way for clinical investigations.

Recent clinical studies have served to help bridge the gap between non-human and human primate basic science by evaluating relationships between maternal immune function and neuroanatomic abnormalities. Maternal pro-inflammatory cytokine interleukin-6 concentrations were associated with offspring frontolimbic white matter microstructural properties, including maturational changes in the first 12 months postnatally (27). Another clinical study linked high maternal inflammatory concentration of interleukin-6, a pro-inflammatory cytokine, during pregnancy with abnormal development in offspring at 2 years of age in brain regions associated with sensory processing and impulse control (28).

## Stress and MIA

Many studies have suggested a correlation between maternal stress during pregnancy and a myriad of negative neurodevelopmental effects in offspring (29, 30). Stressful experiences during pregnancy, including death in the family, war, natural disasters, and more recently socioeconomic disadvantage have been linked with schizophrenia in offspring (31–34). These results provide evidence for an association between maternal stress and schizophrenia in offspring. The impact of prenatal exposure to maternal stress has been investigated by several birth cohort studies. Distressing experiences while the mother was pregnant were recorded and used to anticipate potential risk of psychiatric disorders among offspring in a Danish cohort (35). There is a growing body of evidence implicating stress during prenatal development to ASD (36, 37).These results corroborate epidemiological research on birth cohorts from the Dutch Hunger Winter of 1944–45 which reported relationships between prenatal famine and offspring long-term cognitive and mental health development (38–40), including schizophrenia and affective disorders. Although nutritional deficiency is regarded as the likely cause of the findings, it is possible that maternal stress due to the exposure played a role. However, conflicting results were found in a population-based cohort study, regarding maternal exposure to death of a relative and risk for ASD in offspring, in which no correlation was reported (41).

Although the precise mechanisms for the associations between maternal stress, immune activation, and subsequent offspring pathology are still not well known, it is thought that psychological stress, through the inflammatory response, may exert an influence on human health (42). One study has examined the cytokine profiles of umbilical cord blood, in association with prenatal stress, as a marker of their effects on the immune system. The findings suggest that both adaptive and innate immune responses were altered by prenatal stress (43). A more recent birth cohort investigation implicated maternal psychological stress in alterations of perinatal cytokine profile in offspring. In particular, prenatal maternal stress was associated with higher levels of interleukin-4, interleukin-5, interleukin-6, interleukin-8, and interleukin-1ß (44).

## The Microbiome and MIA

The microbiome is a relatively new topic that has been explored as a potential etiologic factor in central nervous system disorders and the remediation of their symptoms. An imbalance in the microbiome is correlated with a variety of adverse consequences, including lasting behavioral abnormalities, neuropathology, immune dysfunction, and deficient gastrointestinal integrity. Abnormalities in immune function are reported in ASD and other psychiatric disorders, and recent studies suggest that microbiota is an important factor in this dysregulation (45, 46). The gut microbiome composition has been determined to not only be affected by neuroinflammation (46) but to reciprocally affect specific regional immune responses in the brain (47).

### Animal Models

Experimental studies have shown that MIA brings about enduring changes in immune system activity as well as ubiquitous alterations in the balance of offspring microbiota in adulthood (48–50). One study reported changing the microbiome of mice using human commensal *B. fragilis* enhanced not only gastrointestinal health, but also execution of certain tasks used to measure behaviors principally associated with ASD (49). In another study, investigators found that mice that had more Th17 cells in their intestine, and in which there was more colonization with segmented filamentous bacteria (SFB), were more prone to behavioral pathology caused by MIA (50). This susceptibility was passed to other mice by induction of Th17 cells and colonization of SFB. In addition, during MIA, elevated interleukin-17a responses were caused by the activation of dendritic cells, a key cell type involved in CNS pathology, interacting with SFB colonized Th17 cells (50).

### MIA and the Human Microbiome

When the maternal immune system is activated during pregnancy, the inflammatory cytokines released affect the offspring’s vagal system and consequently their CNS regulation (51). MIA activation also affects maternal gut bacteria, which in turn can affect the microbiome of offspring. The microbiome of offspring has been shown to be populated and affected by the prenatal environment (52), mode of delivery (53), diet, and other aspects of postnatal care (54). The microbiome of children with ASD, when compared with controls, is less diverse, with overgrowth of certain microbes, such as *Desulfovibrio* (55), *Alistipes*, and *Akkermansia* (56), being more common.

Probiotics are hypothesized to aid autism symptoms by colonizing the gastrointestinal system with beneficial bacteria. However, clinical trials of probiotic supplementation have shown mixed results for the effectiveness of probiotics on the behavioral symptoms of ASD (57). A more recent open-label study using microbiota transfer therapy (MTT), which consists of round of an antibiotic, a colon cleanse, and fecal transplant therapy, resulted in an 80% decrease in problematic GI symptoms using the Gastrointestinal Symptom Rating Scale and increased diversity of the microbiome of participants (58). This therapy also resulted in improved ASD behavioral symptoms which continued for 8 weeks post-treatment completion. This therapy will need to be studied more extensively with larger sample sizes, but these results are promising for a potential treatment option.

It has recently been reported that oral probiotic supplementation during pregnancy reduced MIA cytokine levels and subsequent offspring ASD symptoms, such as depression, anxiety, and social deficits, in mouse models (59). Some of these results may be due to the prevention of Poly(I:C)‐induced weight loss of dams, another result of the oral probiotic supplementation. Although this has yet to be studied in humans, this offers insight into potential preventative measures for expecting mothers.

## Epigenetics and MIA

It has been found that epigenetic modifications occur beyond early embryonic development and are dynamic throughout fetal development and over one’s lifetime (60, 61). Epigenetic alterations offer possible mechanisms by which immune insults during prenatal development affect offspring outcomes. Maternal distress has been reported to be a leading cause in epigenetic alterations (61). Birth cohort studies investigating the effects of the Dutch Hunger Winter have examined whether standard DNA methylation is modified as a result of maternal famine and stress. Hypo-methylation during gestation alters the accessibility of offspring DNA to translation and therefore changes gene expression in these regions. Several genes, including ABCA1, insulin-like growth factor II, interleukin-10, GNASAS, and MEG3 have been found to have modified levels of DNA methylation in offspring, thus implicating extensive epigenetic effects of maternal famine (62, 63).

In mouse models of MIA, adult offspring have displayed hypo-methylation, and transcriptional changes, in genes related to GABAergic signaling and neural development (64). In a more recent review, maternal depression, and its associated immunological alterations in cytokines and reactive oxygen species levels, was linked to offspring DNA methylation (65). Experimental evidence from animal models has indicated that MIA can result in widespread DNA hypo-methylation in the hypothalamus (66). This can be a potential factor for dysregulation of the hypothalamus–pituitary–adrenal gland (HPA) axis, which has been linked to the pathophysiology of schizophrenia (67). Alterations in the gray-matter composition of the hypothalamus have also been linked to ASD (68). Another study reported that, in MIA exposed mice, 80% of hypo-methylated sites were stabilized with a diet high in anti-inflammatory fats (69). Although this is yet to be studied in humans, this has profound implications for possible dietary interventions to mitigate the effects of MIA induced hypo-methylation in addition to standard treatment.

MIA also alters histone acetylation. Adult female offspring of MIA mice expressed anhedonic behavior, which was correlated with global histone acetylation changes in the hippocampus (70). Histone modification caused by MIA may alter hippocampal serotonin transporter (SERT) expression, a critical component to the etiology of depression and which may play a significant role in schizophrenia (70, 71).

## Future Research and Perspectives

Great strides have been made through both epidemiologic work and basic science to explore the potential role of MIA in neuropsychiatric disorders. The addition of epigenetics to the MIA model as a mediating mechanism may shed more light on pathogenic processes that underlie these disorders. A key challenge regarding a suitable translational approach (12) is the Research Domain Criteria (RDOC) (72), which is aimed at deconstructing psychiatric disorders into their most basic psycho- and neuropathological components. Further insights for future translational research may be gleaned from standardization of immune activating agents and methods, integrating postmortem pathology, and longitudinal neuroimaging (73–75).

Although stress has been conceptualized as a teratogen, and may activate the maternal immune system in a way similar to infection, the biological framework for how it may affect offspring is still not well understood. Beyond cytokines, maternal cortisol levels have also been implicated in offspring neuropathology (76). Further elucidation of the biological mechanism by which maternal stress may act as an inflammatory agent, and influence offspring neuropathology relevant to psychiatry disorders, is necessary.

Although investigation of the microbiome offers the potential for important findings linking the immune system and psychopathology, several issues remain. For example, whether exposure to known risk factors for ASD and other psychiatric outcomes also result in microbiome alterations requires further investigation. Another question of interest relates to the cause-effect relationship between MIA and the maternal microbiota, on offspring neurodevelopment. Studies comparing psychiatric outcomes following C-section versus natural birth creates opportunities to address this question given the differences in exposure to the vaginal microbiome between the two delivery methods (77).

## Conclusions

In conclusion, we propose it is vital to consider MIA in the context of not only infection but also other factors, such as maternal psychological stress, in the etiology of neurodevelopmental disorders. Epigenetic events may represent mediating or modifying factors in the putative pathogenesis of psychiatric disorders following MIA. The microbiome is another promising area of investigation in the MIA hypothesis of mental disorders. We believe that a translational approach that incorporates knowledge of these processes will be necessary to broaden our understanding of the effects of prenatal MIA on offspring susceptibility to psychiatric disorders.

## Author Contributions

FC wrote the first draft of the manuscript. AB contributed to the conception, editing, and research for the manuscript. Both authors contributed to manuscript revision and have read and approved the final manuscript.

## Funding

This manuscript was supported by NIMH R01MH082052 (A.S.B), and by NIEHS R01ES019004 (A.S.B).

## Conflict of Interest Statement

The authors declare that the research was conducted in the absence of any commercial or financial relationships that could be construed as a potential conflict of interest.

## References

[1] Canetta S Sourander A Surcel HM Hinkka-Yli-Salomäki S Leiviskä J Kellendonk C Elevated maternal C-reactive protein and increased risk of schizophrenia in a national birth cohort. Am J Psychiatry (2014) 171:960–8. 10.1176/appi.ajp.2014.13121579 PMC4159178 24969261

[2] Brown AS Derkits EJ Prenatal infection and schizophrenia: a review of epidemiologic and translational studies. Am J Psychiatry (2010) 167:261–80. 10.1176/appi.ajp.2009.09030361 PMC3652286 20123911

[3] Brown AS Sourander A Hinkka-Yli-Salomäki S Mckeague IW Sundvall J Surcel HM Elevated maternal C-reactive protein and autism in a national birth cohort. Mol Psychiatry (2014) 19:259–64. 10.1038/mp.2012.197 PMC3633612 23337946

[4] Patterson PH Xu W Smith SE Devarman BE Maternal immune activation, cytokines and autism. In: Autism. Totowa, NJ: Springer (2008). 10.1007/978-1-60327-489-0_13

[5] Parboosing R Bao Y Shen L Schaefer CA Brown AS Gestational influenza and bipolar disorder in adult offspring. JAMA Psychiatry (2013) 70:677–85. 10.1001/jamapsychiatry.2013.896 23699867

[6] Mednick SA Machon RA Huttunen MO Bonett D Adult schizophrenia following prenatal exposure to an influenza epidemic. Arch Gen Psychiatry (1988) 45:189–92. 10.1001/archpsyc.1988.01800260109013 3337616

[7] Kilbourne ED Influenza. New York, NY: Plenum Medical Book Co. (1987). 10.1007/978-1-4684-5239-6

[8] Debost JP Larsen JT Munk-Olsen T Mortensen PB Meyer U Petersen L Joint effects of exposure to prenatal infection and peripubertal psychological trauma in schizophrenia. Schizophr Bull (2017) 43:171–9. 10.1093/schbul/sbw083 PMC5216853 27343007

[9] Jiang HY Xu LL Shao L Xia RM Yu ZH Ling ZX Maternal infection during pregnancy and risk of autism spectrum disorders: a systematic review and meta-analysis. Brain Behav Immun (2016) 58:165–72. 10.1016/j.bbi.2016.06.005 27287966

[10] Abdallah MW Larsen N Grove J Norgaard-Pedersen B Thorsen P Mortensen EL Amniotic fluid chemokines and autism spectrum disorder: an exploratory study utilizing a Danish Historic Birth Cohort. Brain Behav Immun (2012) 26:170–6. 10.1016/j.bbi.2011.09.003 21933705

[11] Oliveira J Oliveira-Maia AJ Tamouza R AS B Leboyer M Infectious and immunogenetic factors in bipolar disorder. Acta Psychiatr Scand (2017) 136(4):409–23. 10.1111/acps.12791 PMC7159344 28832904

[12] Brown AS Meyer U Maternal immune activation and neuropsychiatric illness: a translational research perspective. Am J Psychiatry (2018) 11:1073–83. 10.1176/appi.ajp.2018.17121311 PMC6408273 30220221

[13] Gilmore JH Jarskog LF Exposure to infection and brain development: cytokines in the pathogenesis of schizophrenia. Schizophr Res (1997) 24:365–7. 10.1016/S0920-9964(96)00123-5 9134598

[14] Patterson PH Neuroscience. Science (2007) 318:576–7. 10.1126/science.1150196 17962542

[15] Burns TM Clough JA Klein RM Wood GW Berman NEJ Developmental regulation of cytokine expression in the mouse brain. Growth Factors (1993) 9:253–8. 10.3109/08977199308991585 8148154

[16] Gayle DA Beloosesky R Desai M Amidi F Nuñez SE Ross MG Maternal LPS induces cytokines in the amniotic fluid and corticotropin releasing hormone in the fetal rat brain. Am J Physiol Regul Integr Comp Physiol (2004) 286:R1024–R1029. 10.1152/ajpregu.00664.2003 14988088

[17] Meyer U Prenatal poly(i:C) exposure and other developmental immune activation models in rodent systems. Biol Psychiatry (2014) 75:307–15. 10.1016/j.biopsych.2013.07.011 23938317

[18] Meyer U Feldon J Fatemi SH In-vivo rodent models for the experimental investigation of prenatal immune activation effects in neurodevelopmental brain disorders. Neurosci Biobehav Rev (2009) 33:1061–79. 10.1016/j.neubiorev.2009.05.001 19442688

[19] Meyer U Feldon J To poly(I:C) or not to poly(I:C): advancing preclinical schizophrenia research through the use of prenatal immune activation models. Neuropharmacol (2012) 62:1308–21. 10.1016/j.neuropharm.2011.01.009 21238465

[20] Khan D Fernando P Cicvaric A Berger A Pollak A Monje FJ Long-term effects of maternal immune activation on depression-like behavior in the mouse. Transl Psychiatry (2014) 4:e363. 10.1038/tp.2013.132 24548878 PMC3944633

[21] Schwartzer JJ Careaga M Coburn MA Rose DR Hughes HK Ashwood P Behavioral impact of maternal allergic-asthma in two genetically distinct mouse strains. Brain Behav Immun (2017) 63:99–107. 10.1016/j.bbi.2016.09.007 27622677 PMC5346064

[22] Malkova NV Yu CZ Hsiao EY Moore MJ Patterson PH Maternal immune activation yields offspring displaying mouse versions of the three core symptoms of autism. Brain Behav Immun (2012) 26:607–16. 10.1016/j.bbi.2012.01.011 PMC3322300 22310922

[23] Giovanoli S Engler H Engler A Richetto J Voget M Willi R Stress in puberty unmasks latent neuropathological consequences of prenatal immune activation in mice. Science (2013) 339:1095–9. 10.1126/science.1228261 23449593

[24] Short SJ Lubach GR Karasin AI Olsen CW Styner M Knickmeyer RC Maternal influenza infection during pregnancy impacts postnatal brain development in the rhesus monkey. Biol Psychiatry (2010) 67:965–73. 10.1016/j.biopsych.2009.11.026 PMC3235476 20079486

[25] Bauman MD Iosif AM Smith SE Bregere C Amaral DG Patterson PH Activation of the maternal immune system during pregnancy alters behavioral development of rhesus monkey offspring. Biol Psychiatry (2014) 75:332–41. 10.1016/j.biopsych.2013.06.025 PMC6782053 24011823

[26] Weir RK Forghany R Smith SE Patterson PH Mcallister AK Schumann CM Preliminary evidence of neuropathology in nonhuman primates prenatally exposed to maternal immune activation. Brain Behav Immun (2015) 48:139–46. 10.1016/j.bbi.2015.03.009 PMC5671487 25816799

[27] Rasmussen JM Graham AM Entringer S Gilmore JH Styner M Fair DA Maternal Interleukin-6 concentration during pregnancy is associated with variation in frontolimbic white matter and cognitive development in early life. Neuroimage (2018) 185:825–35. 10.1016/j.neuroimage.2018.04.020 PMC6181792 29654875

[28] Graham AM Rasmussen JM Rudolph MD Heim CM Gilmore JH Styner M Maternal systemic interleukin-6 during pregnancy is associated with newborn amygdala phenotypes and subsequent behavior at 2 years of age. Biol Psychiatry (2018) 83:109–19. 10.1016/j.biopsych.2017.05.027 PMC5723539 28754515

[29] Class QA Abel KM Khashan AS Rickert ME Dalman C Larsson H Offspring psychopathology following preconception, prenatal and postnatal maternal bereavement stress. Psychol Med (2014) 44:71–84. 10.1017/S0033291713000780 23591021 PMC3766407

[30] Walder DJ Laplante DP Sousa-Pires A Veru F Brunet A King S Prenatal maternal stress predicts autism traits in 6(1/2) year-old children: project Ice Storm. Psychiatry Res (2014) 219:353–60. 10.1016/j.psychres.2014.04.034 24907222

[31] Huttunen MO Niskanen P Prenatal loss of father and psychiatric disorders. Arch Gen Psychiatry (1978) 35:429–31. 10.1001/archpsyc.1978.01770280039004 727894

[32] Watson JB Mednick SA Huttunen M Wang X Prenatal teratogens and the development of adult mental illness. Dev Psychopathol (1999) 11:457–66. 10.1017/S0954579499002151 10532619

[33] Malaspina D Corcoran C Kleinhaus KR Perrin MC Fennig S Nahon D Acute maternal stress in pregnancy and schizophrenia in offspring: a cohort prospective study. BMC Psychiatry (2008) 8:71. 10.1186/1471-244X-8-71 18717990 PMC2546388

[34] Gilman SE Hornig M Ghassabian A Hahn J Cherkerzian S Albert PS Socioeconomic disadvantage, gestational immune activity, and neurodevelopment in early childhood. Proc Natl Acad Sci U S A (2017) 114:6728–33. 10.1073/pnas.1617698114 PMC5495226 28607066

[35] Khashan AS Abel KM Mcnamee R Pedersen MG Webb RT Baker PN Higher risk of offspring schizophrenia following antenatal maternal exposure to severe adverse life events. Arch Gen Psychiatry (2008) 65:146–52. 10.1001/archgenpsychiatry.2007.20 18250252

[36] Beversdorf DQ Manning SE Hillier A Anderson SL Nordgren RE Walters SE Timing of prenatal stressors and autism. J Autism Dev Disord (2005) 35:471–8. 10.1007/s10803-005-5037-8 16134032

[37] Kinney DK Miller AM Crowley DJ Huang E Gerber E Autism prevalence following prenatal exposure to hurricanes and tropical storms in Louisiana. J Autism Dev Disord (2008) 38:481–8. 10.1007/s10803-007-0414-0 17619130

[38] Hoek HW Brown AS Susser E The Dutch famine and schizophrenia spectrum disorders. Soc Psychiatry Psychiatr Epidemiol (1998) 33:373–9. 10.1007/s001270050068 9708024

[39] Brown AS Susser ES Lin SP Neugebauer R Gorman JM Increased risk of affective disorders in males after second trimester prenatal exposure to the Dutch hunger winter of 1944-45. Br J Psychiatry (1995) 166:601–6. 10.1192/bjp.166.5.601 7620744

[40] Brown AS Van OS J Driessens C Hoek HW Susser ES Further evidence of relation between prenatal famine and major affective disorder. Am J Psychiatry (2000) 157:190–5. 10.1176/appi.ajp.157.2.190 10671386

[41] Li J Vestergaard M Obel C Christensen J Precht DH Lu M A nationwide study on the risk of autism after prenatal stress exposure to maternal bereavement. Pediatrics (2009) 123:1102–7. 10.1542/peds.2008-1734 19336368

[42] Glover V Prenatal stress and its effects on the fetus and the child: possible underlying biological mechanisms. Adv Neurobiol (2015) 10:269–83. 10.1007/978-1-4939-1372-5_13 25287545

[43] Wright RJ Visness CM Calatroni A Grayson MH Gold DR Sandel MT Prenatal maternal stress and cord blood innate and adaptive cytokine responses in an inner-city cohort. Am J Respir Crit Care Med (2010) 182:25–33. 10.1164/rccm.200904-0637OC 20194818 PMC2902757

[44] Andersson NW LI Q Mills CW Ly J Nomura Y Chen J Influence of prenatal maternal stress on umbilical cord blood cytokine levels. Arch Womens Ment Health (2016) 19:761–7. 10.1007/s00737-016-0607-7 PMC5032828 26846778

[45] Hsiao EY Immune dysregulation in autism spectrum disorder. Int Rev Neurobiol (2013) 113:269–302. 10.1016/B978-0-12-418700-9.00009-5 24290389

[46] Cryan JF Dinan TG Gut microbiota: microbiota and neuroimmune signalling-Metchnikoff to microglia. Nat Rev Gastroenterol Hepatol (2015) 12:494–6. 10.1038/nrgastro.2015.127 26215386

[47] Erny D Hrabe de Angelis AL Jaitin D Wieghofer P Staszewski O David E Host microbiota constantly control maturation and function of microglia in the CNS. Nat Neurosci (2015) 18:965–77. 10.1038/nn.4030 PMC5528863 26030851

[48] Mandal M Donnelly R Elkabes S Zhang P Davini D David BT Maternal immune stimulation during pregnancy shapes the immunological phenotype of offspring. Brain Behav Immun (2013) 33:33–45. 10.1016/j.bbi.2013.04.012 23643646

[49] Hsiao EY Mcbride SW Hsien S Sharon G Hyde ER Mccue T Microbiota modulate behavioral and physiological abnormalities associated with neurodevelopmental disorders. Cell (2013) 155:1451–63. 10.1016/j.cell.2013.11.024 PMC3897394 24315484

[50] Kim S Kim H Yim YS Ha S Atarashi K Tan TG Maternal gut bacteria promote neurodevelopmental abnormalities in mouse offspring. Nature (2017) 549:528–32. 10.1038/nature23910 PMC5870873 28902840

[51] Yarandi SS Peterson DA Treisman GJ Moran TH Pasricha PJ Modulatory effects of gut microbiota on the central nervous system: how gut could play a role in neuropsychiatric health and diseases. J Neurogastroenterol Motil (2016) 22:201–12. 10.5056/jnm15146 PMC4819858 27032544

[52] Jimenez E Marin ML Martin R Odriozola JM Olivares M Xaus J Is meconium from healthy newborns actually sterile? Res Microbiol (2008) 159:187–93. 10.1016/j.resmic.2007.12.007 18281199

[53] Bokulich NA Chung J Battaglia T Henderson N Jay M Li H A DL Antibiotics, birth mode, and diet shape microbiome maturation during early life. Sci Transl Med (2016) 8:343ra82. 10.1126/scitranslmed.aad7121 PMC5308924 27306664

[54] Yassour M Vatanen T Siljander H Hamalainen AM Harkonen T Ryhanen SJ Natural history of the infant gut microbiome and impact of antibiotic treatment on bacterial strain diversity and stability. Sci Transl Med (2016) 8:343ra81. 10.1126/scitranslmed.aad0917 PMC5032909 27306663

[55] Finegold SM Desulfovibrio species are potentially important in regressive autism. Med Hypotheses (2011) 77:270–4. 10.1016/j.mehy.2011.04.032 21592674

[56] de Angelis M Piccolo M Vannini L Siragusa S de Giacomo A Serrazzanetti DI Fecal microbiota and metabolome of children with autism and pervasive developmental disorder not otherwise specified. PLoS One (2013) 8:e76993. 10.1371/journal.pone.0076993 24130822 PMC3793965

[57] Navarro F Liu Y Rhoads JM Can probiotics benefit children with autism spectrum disorders? World J Gastroenterol (2016) 22:10093–102. 10.3748/wjg.v22.i46.10093 PMC5155168 28028357

[58] Kang DW Adams JB Gregory AC Borody T Chittick L Fasano A Microbiota transfer therapy alters gut ecosystem and improves gastrointestinal and autism symptoms: an open-label study. Microbiome (2017) 5:10. 10.1186/s40168-016-0225-7 28122648 PMC5264285

[59] Wang X Yang J Zhang H Yu J Yao Z Oral probiotic administration during pregnancy prevents autism-related behaviors in offspring induced by maternal immune activation via anti-inflammation in mice. Autism Res (2019) 12:576–88. 10.1002/aur.2079 30681777

[60] Champagne FA Epigenetic influence of social experiences across the lifespan. Dev Psychobiol (2010) 52:299–311. 10.1002/dev.20436 20175106

[61] Monk C Spicer J Champagne FA Linking prenatal maternal adversity to developmental outcomes in infants: the role of epigenetic pathways. Dev Psychopathol (2012) 24:1361–76. 10.1017/S0954579412000764 PMC3730125 23062303

[62] Heijmans BT Tobi EW Stein AD Putter H Blauw GJ Susser ES Persistent epigenetic differences associated with prenatal exposure to famine in humans. Proc Natl Acad Sci U S A (2008) 105:17046–9. 10.1073/pnas.0806560105 PMC2579375 18955703

[63] Tobi EW Lumey LH Talens RP Kremer D Putter H Stein AD DNA methylation differences after exposure to prenatal famine are common and timing- and sex-specific. Hum Mol Genet (2009) 18:4046–53. 10.1093/hmg/ddp353 PMC2758137 19656776

[64] Richetto J Massart R Weber-Stadlbauer U Szyf M Riva MA Meyer U Genome-wide DNA Methylation Changes in a Mouse Model of Infection-Mediated Neurodevelopmental Disorders. Biol Psychiatry (2017) 81:265–76. 10.1016/j.biopsych.2016.08.010 27769567

[65] Rakers F Rupprecht S Dreiling M Bergmeier C Witte OW Schwab M Transfer of maternal psychosocial stress to the fetus. Neuroscience & Biobehavioral Reviews (2017) S0149-7635(16)30719-9. 10.1016/j.neubiorev.2017.02.019 28237726

[66] Basil P Li Q Dempster EL Mill J Sham PC Wong CC Prenatal maternal immune activation causes epigenetic differences in adolescent mouse brain. Transl Psychiatry (2014) 4:e434. 10.1038/tp.2014.80 25180573 PMC4203009

[67] Tognin S Rambaldelli G Perlini C Bellani M Marinelli V Zoccatelli G Enlarged hypothalamic volumes in schizophrenia. Psychiatry Res (2012) 204:75–81. 10.1016/j.pscychresns.2012.10.006 23217575

[68] Kurth F Narr KL Woods RP O’neill J Alger JR Caplan R Diminished gray matter within the hypothalamus in autism disorder: a potential link to hormonal effects? Biol Psychiatry (2011) 70:278–82. 10.1016/j.biopsych.2011.03.026 PMC3134572 21531390

[69] Basil P Li Q Gui H Hui TCK Ling VHM Wong CCY Prenatal immune activation alters the adult neural epigenome but can be partly stabilised by a n-3 polyunsaturated fatty acid diet. Translational Psychiatry (2018) 8:125. 10.1038/s41398-018-0167-x 29967385 PMC6028639

[70] Reisinger SN Kong E Khan D Schulz S Ronovsky M Berger S Maternal immune activation epigenetically regulates hippocampal serotonin transporter levels. Neurobiol Stress (2016) 4:34–43. 10.1016/j.ynstr.2016.02.007 27981188 PMC5146201

[71] Li W Yang Y Lin J Wang S Zhao J Yang G Association of serotonin transporter gene (SLC6A4) polymorphisms with schizophrenia susceptibility and symptoms in a Chinese-Han population. Prog Neuropsychopharmacol Biol Psychiatry (2013) 44:290–5. 10.1016/j.pnpbp.2013.04.003 23583772

[72] Insel T Cuthbert B Garvey M Heinssen R Pine DS Quinn K Research domain criteria (RDoC): toward a new classification framework for research on mental disorders. Am J Psychiatry (2010) 167:748–51. 10.1176/appi.ajp.2010.09091379 20595427

[73] Careaga M Murai T Bauman MD Maternal immune activation and autism spectrum disorder: from rodents to nonhuman and human primates. Biol Psychiatry (2017) 81:391–401. 10.1016/j.biopsych.2016.10.020 28137374 PMC5513502

[74] Bauman MD Schumann CM Is ‘bench-to-bedside’ realistic for autism? An integrative neuroscience approach. Neuropsychiatry (London) (2013) 3:159–68. 10.2217/npy.13.18 PMC3757943 24000295

[75] Kentner AC Bilbo SD Brown AS Hsiao EY Mcallister AK Meyer U Maternal immune activation: reporting guidelines to improve the rigor, reproducibility, and transparency of the model. Neuropsychopharmacology (2018) 44:245–58. 10.1038/s41386-018-0185-7 PMC6300528 30188509

[76] Ellman LM Schetter CD Hobel CJ Chicz-Demet A Glynn LM Sandman CA Timing of fetal exposure to stress hormones: effects on newborn physical and neuromuscular maturation. Dev Psychobiol (2008) 50:232–41. 10.1002/dev.20293 PMC2851937 18335490

[77] Jasarevic E Howerton CL Howard CD Bale TL Alterations in the vaginal microbiome by maternal stress are associated with metabolic reprogramming of the offspring gut and brain. Endocrinology (2015) 156:3265–76. 10.1210/en.2015-1177 PMC4541625 26079804

